# Dual ACE2 epitope-based biomimetic receptors for selective sensing of SARS-CoV variants

**DOI:** 10.1038/s41598-025-20837-6

**Published:** 2025-09-23

**Authors:** Tiba Al-Dujaili, Sara Björk Sigurdardóttir, Verónica A. Jiménez, Michele Larocca, Börje Sellergren

**Affiliations:** 1 Department of Biomedical Sciences and Biofilms-Research Center for Biointerfaces (BRCB), Faculty of Health and Society, Malmö University, 20506 Malmö, Sweden; 2 Departamento de Ciencias Químicas, Facultad de Ciencias Exactas, Universidad Andres Bello, Autopista Concepción-Talcahuano, 7100 Talcahuano, Chile

**Keywords:** Biosensor, Combinatorial detection, SARS-CoV-2, SARS-CoV variants, SPR, ACE2-based sensor, Biochemistry, Biophysics, Biotechnology, Computational biology and bioinformatics, Microbiology

## Abstract

We report a combinatorial approach to design peptide-based biomimetic sensors for detecting β-type coronaviruses with high sensitivity and selectivity. We selected three peptide epitopes from key regions of the ACE2 receptor that are involved in viral binding to different variants and immobilized them individually or in binary combinations on gold sensor chips. Using Surface Plasmon Resonance (SPR), we found that single-epitope sensors displayed nanomolar dissociation constants to three RBD variants (SARS-CoV-2 Delta < SARS-CoV-2 Alpha < SARS-CoV-1) and a K_D_ = 1.2 ± 0.4 nM to the full SARS-CoV-2 Alpha spike protein, with negligible binding to the a-coronavirus hCoV-NL63 spike protein. Molecular dynamics simulations revealed that the tightest binding epitope closely mimics ACE2 interactions with β-coronaviruses, explaining its superior performance. In contrast, dual epitope systems exhibited a reversed variant preference, with a pronounced affinity enhancement for SARS-CoV-1 (K_D_ = 6 ± 2 nM). This was attributed to cooperative epitope interactions that could restrict the conformational flexibility of the longer epitope, favoring the effective intermolecular contacts that strengthen the interaction with the RBD. These findings suggest a time-saving approach for developing sensitive and selective sensors for rapidly mutating viruses.

**Supplementary Information:**

The online version contains supplementary material available at 10.1038/s41598-025-20837-6.

By early 2020, the coronavirus disease COVID-19 had been declared a pandemic. The coronavirus SARS-CoV-2 was identified as the cause of this severe respiratory disease, which claimed over 1.8 million lives by the end of that year^1,2^. Since then, numerous coronavirus variants have been identified, some with increased transmissibility, constituting a major threat to public health worldwide and claiming a total of 7.05 million deaths as of June 2024^3^. The variants often contain multiple mutations that escape monoclonal antibodies targeting the SARS-CoV-2 spike or neutralizing polyclonal sera, compromising vaccine efficacy. Due to the lag time before robust PCR and antigen tests are available, detecting these mutations is time-consuming and often involves sequencing kilobases of DNA in multiple patients. Moreover, early versions of the rapid antibody-based tests lacked sensitivity for the virus and demonstrated high false-negative rates in patient populations with milder symptoms^4–6^. In this scenario, rapid, sensitive, readily adjustable, and low-cost tests developed within a short time period are in high demand and could make a difference in the preparation for and combating of future pandemics.

Biomimetic receptors have emerged as promising tools for future viral diagnostics, tests, and sensor development^7^. Being designed to mimic the molecular recognition abilities of biological receptors, such as enzymes, antibodies, or membrane proteins, these receptors can selectively bind to target molecules, enabling the development of low-cost and robust sensors or tests^5,7,9^. In line with this challenge, herein we report a biomimetic combinatorial approach to engineer receptors for β-type coronaviral variants based on known contacts determined from early reported cryo-EM data^9^. To this end, three epitopes (**a1**, **a2**, and **rc3**) were strategically chosen based on their distinctive interactions with the Receptor Binding Domain (RBD) of the Spike protein from SARS-CoV-2^9^. The epitope **a1** (residues S19 to N52) is part of the peptidase domain of ACE2 and contains the key residues Q24, T27, D30, K31, H34, E35, E37, D38, Y41, and Q42, that have been reported to play a key role in the formation of the ACE2 complex with the RBD of SARS-CoV-2^9–11^. Epitope **a2** (residues Q76 to L95) is part of the α2-helix of ACE2 and includes residues L79, M82 and Y83, which also play a relevant role in RBD recognition^9,11^. The third epitope, the random coil **rc3** (residues M323 to T334) encompasses residues Q325, E329, and N330, which interact with the RBD of SARS-CoV-1. Modeling studies suggest its importance in enhancing the overall ACE2-RBD binding energy, and the **rc3** sequence has been used in previous inhibitor designs^9–11^. The full contact list between the chosen epitopes and known variants of SARS-CoV-2 based on published cryo-EM results is provided as Supporting Information. The selected peptides were synthesized with an additional propargylglycine as a bioorthogonal site for a copper-catalyzed azide-alkyne cycloaddition (CuAAC) and immobilized on SPR gold sensor chips to achieve single and dual peptide sensors. The binding affinities of each epitope to the Receptor Binding Domains (RBD) of three virus variants and the SARS-CoV-2 Alpha Spike protein were measured using surface plasmon resonance (SPR) followed by modeling of the interactions by dynamics simulations. The results show that RBD affinity and variant specificity can be adjusted based on a judicious choice of combinations of host cell receptor epitopes (Fig. 1). This suggests that tightly binding multivalent receptors for the respective mutants can be engineered by excluding sequences featuring deleted or suppressed contacts while including sequences containing new contacts.

**Fig. 1:**
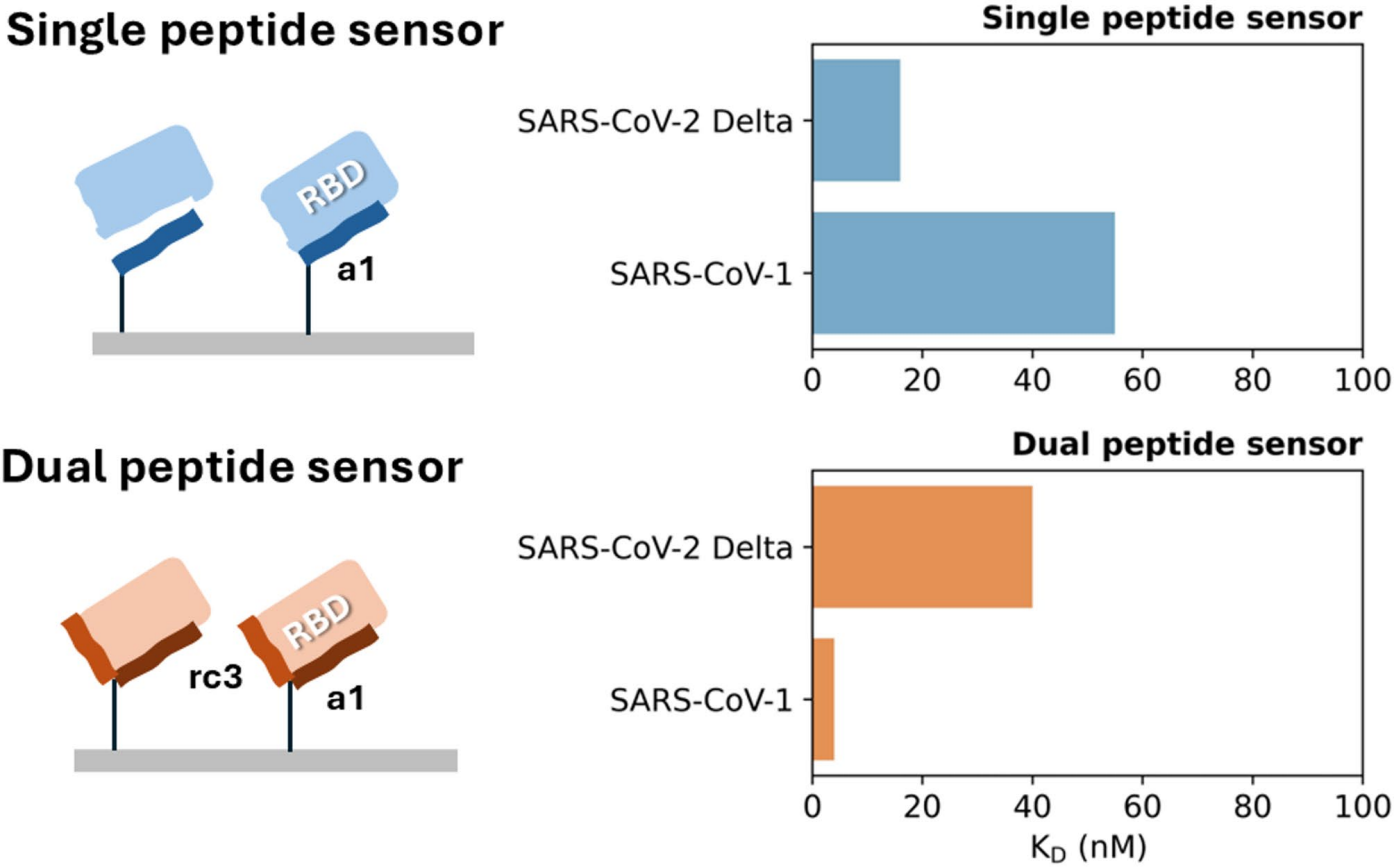
Switching of coronavirus variant preference using different combinations of ACE2 epitopes.

## Results and discussion

This work reports on the rational development of single and dual ACE2 peptide-based biomimetic receptors for the high-affinity detection of SARS-CoV variants. The first stage of this development focused on optimizing the immobilization of peptides onto the surface of SPR gold sensors. Subsequently, the performance of sensors employing single or double epitopes for recognizing the RBD of three β-type variants (SARS-CoV-2 Alpha, SARS-CoV-2 Delta and SARS-CoV-1) and the full spike protein of SARS-CoV-2 Alpha was evaluated. The resulting affinities were rationalized by molecular dynamics simulations of the corresponding ligand-receptor pairs. Detailed information is provided below.

## Peptide immobilization and ligand density tuning

Peptide immobilization was done via a copper-catalyzed cycloaddition reaction, by coupling the ethynyl group of the C-terminal propargylglycine residue to azide-modified SPR gold chips (Xantec AZD-50 L) (Fig. S1). These chips feature a 50 nm thick, low-coverage azide-derivatized carboxymethyldextran hydrogel layer that provides the mobility and flexibility required to ensure high accessibility for the conjugated peptide combined with low nonspecific binding. Although copper-catalyzed click couplings are widely used in organic synthesis and bioconjugations, they are not standard immobilization methods for SPR and, therefore, need optimization. To this end, we conducted pH scouting experiments to determine the optimal acidity conditions for maximizing peptide immobilization efficacy (Supporting Information). Our findings revealed that optimal conditions for peptide immobilization are achieved at pH values near the isoelectric point (pI) of each peptide. This aligns with previous reports^12^ and can be explained by electrostatic considerations. Whereas a higher pH will promote electrostatic repulsion between the negatively charged peptides and the polyanionic carboxylated dextran 3D matrix, lowering the pH below the pK_a_ of the surface carboxylic acids will suppress binding due to a decrease in surface charge density. Acetate buffer was chosen to conduct the immobilization assays to avoid copper sulfate precipitation in the sensitive microfluidic system of the SPR instrument. For binary mixtures (**a1/rc3** and **a2/rc3**), a 1:1 ratio of the respective peptides was prepared in acetate buffer at pH 5. After pH scouting experiments were conducted, it was found that a pH that lies in the middle of the optimal pH range for the individual peptides provided the best results in terms of immobilization efficiency (Figure S2). Using optimized conditions, the peptides were immobilized individually or in binary mixtures, and the immobilization ratio was estimated from the change in response units (RUs) as detailed in Table 1.

**Table 1:** Single and dual epitope immobilization results expressed in both change in response unit (ΔRU) and number of immobilized molecules per mm^2^ (N) of the epitopes from two different sets of experiments on two different SPR chips (± CV). Details on the calculations are provided as supporting Information.

Immobilized peptides	pH of immobilization	ΔRU	*N* × 10^–10^
a1	4	138	2.1 ± 0.6
a2	6.5	880	21 ± 2
rc3	1	307	12 ± 4
a1/rc3	5	802	17 ± 8
a2/rc3	5	550	16 ± 4

Our results showed that immobilization of **a2** resulted in the highest surface coverage, approximately 10 times higher than **a1** and nearly twice that of **rc3** (Supporting Information). For the dual epitope combinations, the highest immobilization efficacy was achieved by the **a1/rc3**, with an 8-fold increase compared to the single immobilization of **a1**. On the other hand, the **a2/rc3** mixture resulted in a diminished immobilization performance compared with **a2**. The differences in immobilization efficiency between single- and double-peptide immobilizations may be influenced by the different isoelectric points, molecular weights, and intermolecular interactions between the mixed peptides. This last aspect was examined from molecular dynamics simulations in binary mixtures (*vide infra*).

### Binding affinity of single peptide sensors to RBD variants

**Fig. 2:**
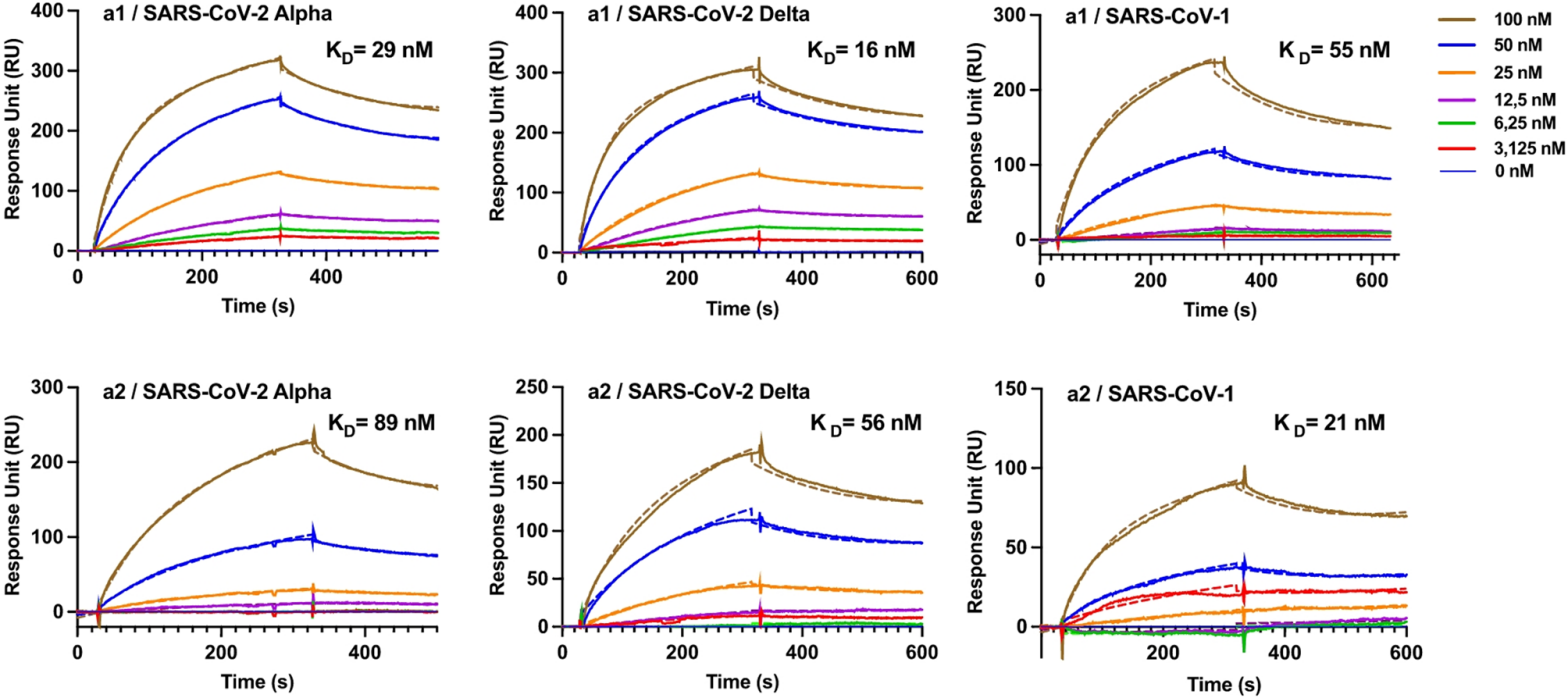
Representative SPR sensorgrams for the binding of three β-type coronavirus RBD variants (SARS-CoV-2 Alpha, SARS-CoV-2 Delta, and SARS-CoV-1) to the immobilized peptides **a1** and **a2**. Fitted curves are inserted as dashed lines.

The single-peptide sensors were tested for their binding affinity to the receptor-binding domain (RBD) of three β-coronavirus proteins, namely SARS-CoV-2 Alpha, SARS-CoV-2 Delta, and SARS-CoV-1, using multi-cycle kinetic experiments. In these tests, multiple analyte injections are performed over the same sensor surface, and after each injection, the surface is regenerated to remove any remaining analyte or complex, allowing for a fresh interaction in subsequent cycles. This approach is useful when the binding kinetics are complex. Our multi-cycle kinetic experiments used five sequential RBD concentrations ranging from 3.1 to 100 nM. Figure 2 shows the SPR sensorgrams for the interaction between the β-type corona RBD variants and the single peptide sensors obtained from **a1** and **a2**.

Sensorgrams were recorded and were globally fitted to a 1:1 Langmuir model^12^. Fit quality was assessed primarily by the Chi² values reported by BIAevaluation and complemented by calculating the root mean squared error (RMSE) in response units (RU) and the coefficient of determination (R²) from the exported traces (Table S6). The 1:1 Langmuir kinetic model was chosen and kept consistent for all sensograms because it provides the standard framework for describing the peptide–protein interactions across all variants and sensor surfaces. We note, however, that the 1:1 model alone has inherent limitations, as it does not explicitly account for mass-transport effects at higher ligand densities, heterogeneity in peptide presentation, or possible rebinding events during dissociation. These limitations were not apparent for the Alpha variant, where the fitted curves closely overlapped with the experimental data. By contrast, the Delta-variant and SARS-CoV-1 sensograms adheared less well to the model suggesting a more heterogeneous interaction with the peptide surface. These deviations were most pronounced on the **a2** sensor, which is possibly linked to the higher immobilization densities of these sensors (Table 1) that in turn could impact ligand conformation and access. Despite these modest discrepancies, the overall χ² values remained low, and the extracted kinetic parameters (Table S7) and K_D_ ranking (Table 2) were robust and in agreement with the molecular dynamics analysis.

The kinetic data and individual dissociation constants for each run are specified in the Supporting Information. A comparative analysis of the binding interactions between the **a1-** or **a2**-based sensors and various RBD variants revealed concentration-dependent analytical responses and K_D_ values in the nanomolar range (16–89 nM) but with significant differences between the sensors. The highest affinity was observed using the **a1** sensor, which showed a binding preference in the order SARS-CoV-2 Delta > SARS-CoV-2 Alpha > SARS-CoV-1. The tighter binding of the Delta variant aligns with other reports and reflects the selection pressure towards mutants displaying a higher affinity for ACE2 and, in turn, infectivity. For **a2**, the relative binding affinities between the SARS-CoV-1 and SARS-CoV-2 variants were reversed with SARS-CoV-1 now showing the strongest binding with a K_D_ of 21 nM. Among the SARS-CoV-2 variants, the Delta RBD displayed the highest affinity also in this case. Data corresponding to **rc3** is provided as Supporting Information, as only weak interactions were detected between the sensors containing this peptide and all RBDs.

**Table 2:** Results from SPR-based kinetic interaction experiments for the interaction between β-type coronaviral variants (SARS-CoV-2 Alpha, SARS-CoV-2 Delta, and SARS-CoV-1) RBDs and Spike proteins and the immobilized peptides a1 and a2.

Immobilized peptide	RBD variant	K_D_ (nM)^a^	RU_max_
a1	SARS-CoV-2 Alpha	29 ± 6	271
	SARS-CoV-2 Delta	16 ± 1	246
	SARS-CoV-1	55 ± 4	260
	SARS-CoV-2 spike	1.2 ± 0.4	605
a2	SARS-CoV-2 Alpha	89 ± 5	277
	SARS-CoV-2 Delta	56 ± 6	204
	SARS-CoV-1	21 ± 5	271
	SARS-CoV-2 spike	19 ± 10	123

^a^Dissociation constant (K_D_) indicates mean ± SD of three independent replicates.

### Binding affinity of single peptide sensors to the full Spike protein of SARS-CoV-2

**Fig. 3:**
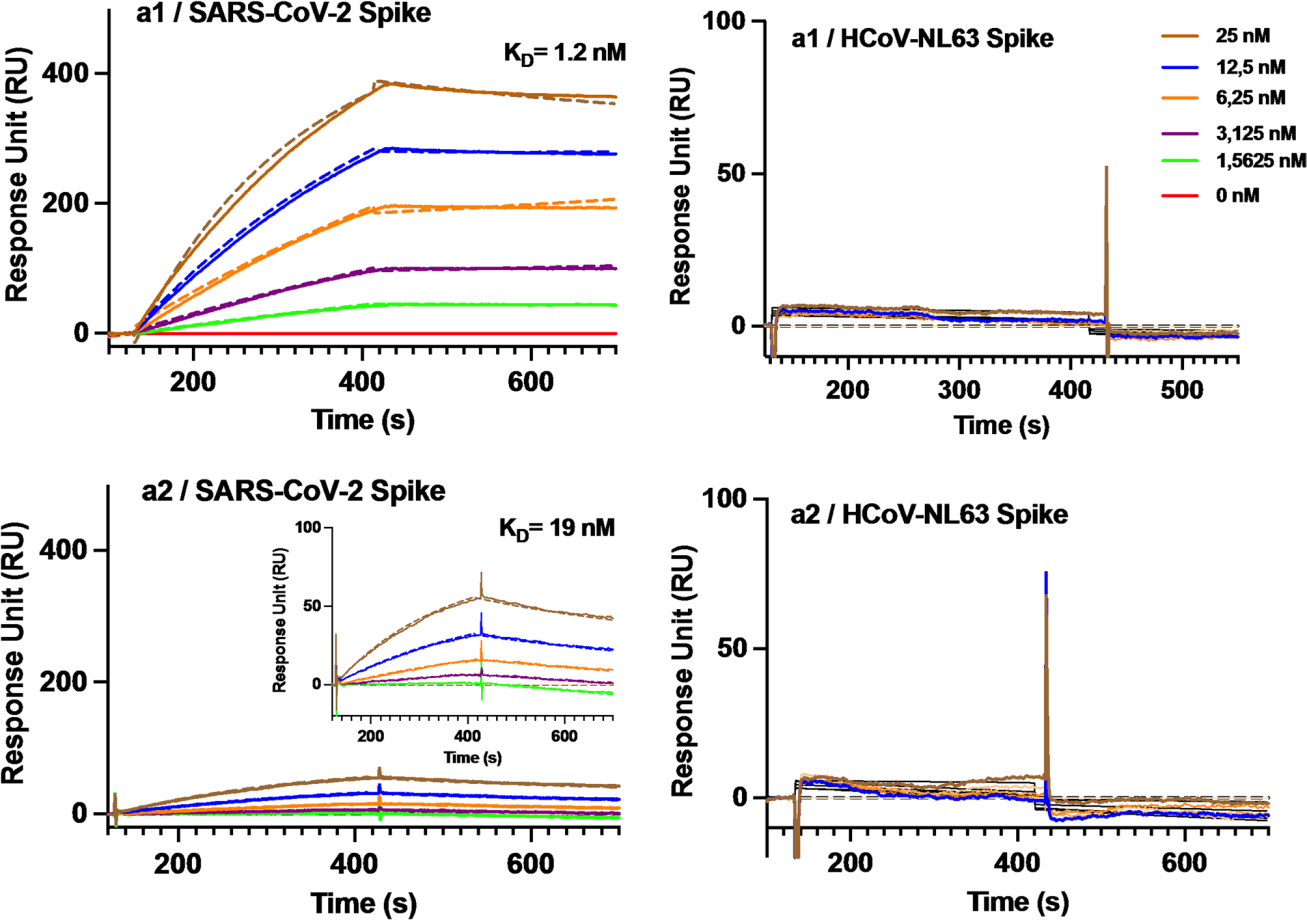
Representative SPR sensorgrams for the binding of the full Spike proteins of SARS-CoV-2 and HCoV-NL63 on sensors containing the immobilized ACE2 peptides **a1** and **a2**. Fitted curves are inserted as dashed lines.

Additional SPR experiments were conducted to test the capacity of the immobilized peptides (**a1**, **a2**, and r**c3**) to recognize the full spike protein of SARS-CoV-2 (Fig. 3; Table 2, Table S8,). Our findings revealed that **a1** binds to the full spike protein with detectable SPR signals even at low nanomolar concentrations, yielding a K_D_ of 1.2 nM and a RU_max_ of 605, which indicates a very high affinity and significant binding capacity. The measured dissociation constant is significantly lower than the estimated values for other sensors targeting the SARS-CoV-2 spike protein and comparable to those of available antibodies for this protein. Additionally, the interaction with the full spike protein is stronger than the interaction with the RBDs, possibly reflecting multivalent interactions due to the trimeric nature of the protein (Table 2). Turning to **a2**, this epitope showed a K_D_ of 19 nM with the full spike protein of SARS-CoV-2 and an RU_max_ of 123, reflecting weaker binding than **a1**, although again stronger than that observed between **a2** and SARS-CoV-2 Alpha RBD. Again, the **rc3** peptide did not show measurable signals attributable to effective interactions with the spike protein, as expected from its negligible interaction with the RBD (data provided as Supporting Information). The selectivity of **a1** and **a2** for SARS-CoV-2 was evaluated by testing the binding of the immobilized epitopes to the spike protein of HCoV-NL63, an a-type coronaviral variant that primarily targets the upper respiratory tract (URT) and induces milder symptoms in comparison to beta coronaviruses such as fever, cough, runny nose, and difficulty breathing^13–17^. SPR experiments confirmed the absence of measurable signals for the interaction of **a1** and **a2** with this protein, underscoring the selectivity of the tested epitopes for β-type coronaviruses.

### Binding affinity of dual epitope sensors SARS-CoV-1 RBD

**Fig. 4:**
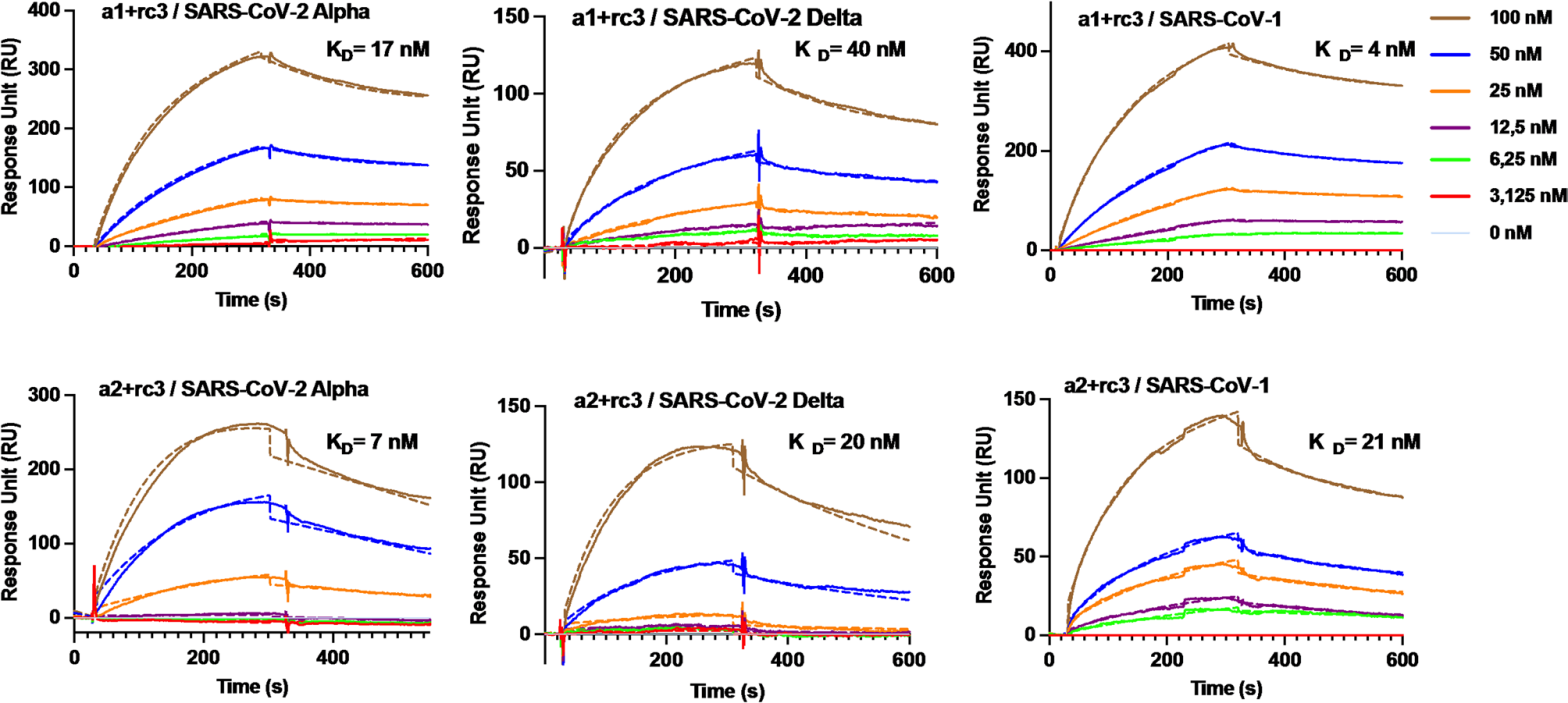
SPR sensorgrams for the binding of three β-type coronaviral variants (SARS-CoV-2 Alpha, SARS-CoV-2 Delta and SARS-CoV-1) RBDs with mixed epitopes **a1** and **a2** with **rc3.** Fitted curves are inserted as dotted lines.

Noticing the different binding preferences of the single epitope receptors, we investigated dual epitope systems to explore whether epitope combinations could also influence the variant selectivity (Fig. 4; Table 3, Table S9). Binary mixtures **a1/rc3** and **a2/rc3** were immobilized on SPR gold chips using equimolar peptide mixtures in acetate buffer (pH 5) to combine the small, random-coil conformation epitope **rc3** with the longer epitopes from the primary ACE2 binding region. SPR experiments revealed that the dual sensors achieved remarkable improvements in binding affinity for SARS-CoV-1 RBD (Fig. 4; Table 3). Notably, the dual sensor **a1/rc3** showed a K_D_​ of 6 nM, marking a 9-fold enhancement compared to the single **a1** sensor, while the sensor **a2/rc3** with a K_D_​= 21 nM was not affected by the presence of **rc3**.

For SARS-CoV-2 Alpha RBD, **a1/rc3** and **a2/rc3** yielded K_D_​ values of 17 nM and 22 nM, respectively, representing 1.7-fold and 4-fold improvements over their single counterparts (K_D_​= 29 nM for **a1** and K_D_ = 89 nM for **a2**). Interestingly, while single sensors based on **a1** exhibited the highest affinity for SARS-CoV-2 Delta and the weakest for SARS-CoV-1 (K_D_​ = 16 nM for Delta and 55 nM for SARS-CoV-1), the dual sensors reversed this trend.

These results underscore the cooperative advantage provided by dual sensors in improving affinity, particularly for challenging targets. The **a1/rc3** dual epitopes consistently demonstrated superior binding affinity across tested variants, while **a2/rc3** showed dramatic improvements relative to single **a2**. The reversal in binding preference between variants highlights how epitope combination influences binding behavior, potentially offering a strategy to tune sensor selectivity. Collectively, these findings support the potential of multivalent interactions in optimizing receptor-based detection and targeting strategies.

**Table 3:** Results from SPR-based kinetic interaction experiments for the interaction between β-type coronaviral variants (SARS-CoV-2 Alpha, SARS-CoV-2 Delta, and SARS-CoV-1) RBDs and the dual epitope sensors based on a1, a2 and rc3.

Immobilized peptide	RBD variant	K_D_ (nM)^a^	RU_max_
a1/rc3	SARS-CoV-2 Alpha	17 ± 1	264
	SARS-CoV-2 Delta	40 ± 2	115
	SARS-CoV-1	6 ± 2	432
a2/rc3	SARS-CoV-2 Alpha	22 ± 2	306
	SARS-CoV-2 Delta	20 ± 1	155
	SARS-CoV-1	21 ± 3	254

^a^Dissociation constant (K_D_) indicates mean ± SD of three independent replicates.

### Molecular dynamics simulations

Molecular Dynamics (MD) simulations were conducted to gain molecular-level insight into our SPR findings regarding the binding affinity of single and dual epitope sensors to β-type coronaviral RBD variants. First, we examined the binding properties of the three peptide epitopes (**a1**, **a2**, and **rc3**) to the RBDs of SARS-CoV-2 Alpha, SARS-CoV-2 Delta, and SARS-CoV-1 to evaluate whether the isolated peptides, modified at the C-termini with the linker moiety that connects the amino acid sequence to the hydrogel layer in the SPR sensor (Figure S3), maintain their conformation and recognition capacity for the binding domains. An a-type variant (HCoV-NL63) was also modeled as a negative control to evaluate the peptides’ selectivity toward β-coronavirus.

**Fig. 5:**
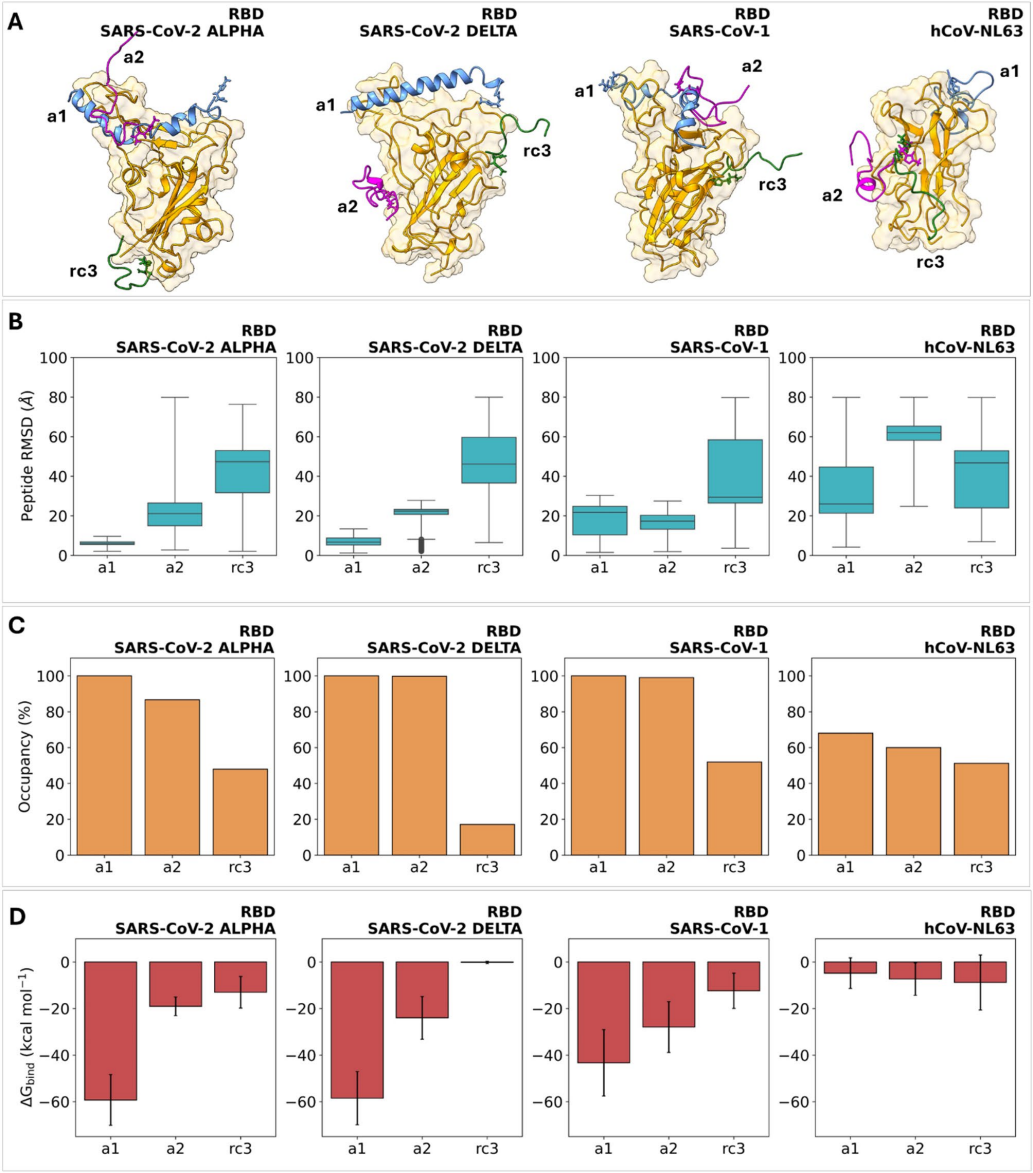
**A** Pictorial representation of the preferred interaction regions for the peptide-RBD complexes formed by **a1**, **a2**, and **rc3** with the RBD domains of SARS-CoV-2 Alpha, SARS-CoV-2 Delta, and SARS-CoV-1 and hCoV-NL63. Images were obtained from 500 ns MD trajectories for single-peptide complexes with the RBDs, considering the most frequent regions of interaction for each peptide. The structures of the complexes for each RBD are superimposed to illustrate the distinct binding modes exhibited by each peptide. **B** RMSD distribution data for peptides **a1**, **a2**, and **rc3** in single-peptide complexes with the RBDs of SARS-CoV-2 Alpha, SARS-CoV-2 Delta, and SARS-CoV-1 and hCoV-NL63. **C** Occupancy of the peptide-protein complexes formed by **a1**, **a2**, and **rc3** and the RBDs tested in this study. **D** Binding free energy estimates (kcal/mol) for the peptide-RBD complexes addressed in this study. MM/GBSA calculations were carried out on 1000 MD frames retrieved from 500 ns MD simulations.

Trajectory analysis revealed that the peptides explore distinct conformations and binding regions on the RBDs. Only **a1** maintained binding modes and conformations similar to those of the epitope in ACE2. In contrast, **a2** and **rc3** showed greater mobility and bound to diverse regions on the RBD, including areas not available for peptide interaction in the full-length spike protein context (Figure S4). Figure 5A provides a pictorial summary of the simulation results, showing the most frequently observed association regions for each of the studied systems. Our results indicate that **a1** binds to the recognition region in all β-type RBDs, whereas **a2** recognizes this region only in the complex with SARS-CoV-1 RBD. Peptide RMSD calculations in protein-aligned MD trajectories align with these observations, showing that **a1** has very low mobility in its complexes with SARS-CoV-2 Alpha and SARS-CoV-2 Delta RBDs (Fig. 5B). This suggests the formation of stable interactions with these receptors, which is consistent with the lower affinity constants measured for these systems by SPR (Table 2). For **a2**, the lowest peptide mobility is observed in the complex with SARS-CoV-1 RBD, which also aligns with our experimental results. For **rc3**, large mobilities are observed in all b-type RBDs, evidencing loose contacts with the proteins. High mobilities were also observed in all three complexes with hCoV-NL63, which is consistent with the minimal binding affinity of the peptides for this receptor detected by SPR (Fig. 3). Additionally, we evaluated the occupancy of the peptide-RBD complexes to assess the permanence of intermolecular contacts in each system (Fig. 5C). Occupancy is defined as the fraction of MD frames in which the peptide interacts with the protein through at least one intermolecular contact at a distance equal to or less than 5 Å. Complexes with **a1** and **a2** have occupancies greater than 80% across all three β-types RBDs, while **rc3** shows significantly lower complex occupancies (< 40%). This aligns with the minimal SPR response observed experimentally for complexes formed by this latter peptide (Supporting Information).

To estimate the strength of the peptide-RBD interactions, we used Molecular Mechanics/Generalized Born Surface Area (MM/GBSA) calculations. MM/GBSA is a well-established computational technique to estimate the binding free energy of a supramolecular complex^18–22^. The free energy difference between the bound and unbound states is calculated using an end-point strategy from the contributions of the molecular mechanics energy (comprising both intra- and intermolecular components) and the solvation free energy (which includes both polar and non-polar terms). While the entropy of the process can be calculated, it’s often omitted under the assumption that systems of similar size and structure, like our single-peptide complexes with various RBDs, should have comparable entropic contributions. While not as rigorous as more computationally demanding methods like alchemical free energy calculations, MM/GBSA offers a powerful balance of computational efficiency and predictive accuracy, making it a valuable tool for understanding and predicting the interactions within peptide-protein systems. Figure 5D summarizes the results of MM/GBSA binding free energy estimates for the series of peptide-RBD complexes under study. For **a1**, the MM/GBSA results show a higher affinity for the SARS-CoV-2 Alpha and Delta variants, which align with our SPR experiments. For **a2**, the results are also consistent with the experimental data, showing a preference for association with the SARS-CoV-1 RBD. Additionally, the peptides show negligible interaction energies with the RBD of hCoV-NL63, accounting for their selectivity to b-type variants. The qualitative agreement between our theoretical and experimental results suggests that our computational models, which are based on interactions between free peptides and receptors, reflect the interactions that would occur in the context of the SPR sensor. This is likely due to the high conformational flexibility of the polymer chains that link the peptide to the chip’s metallic surface, which allows the immobilized peptide to interact with the receptor in a manner very similar to its unbound state.

**Fig. 6:**
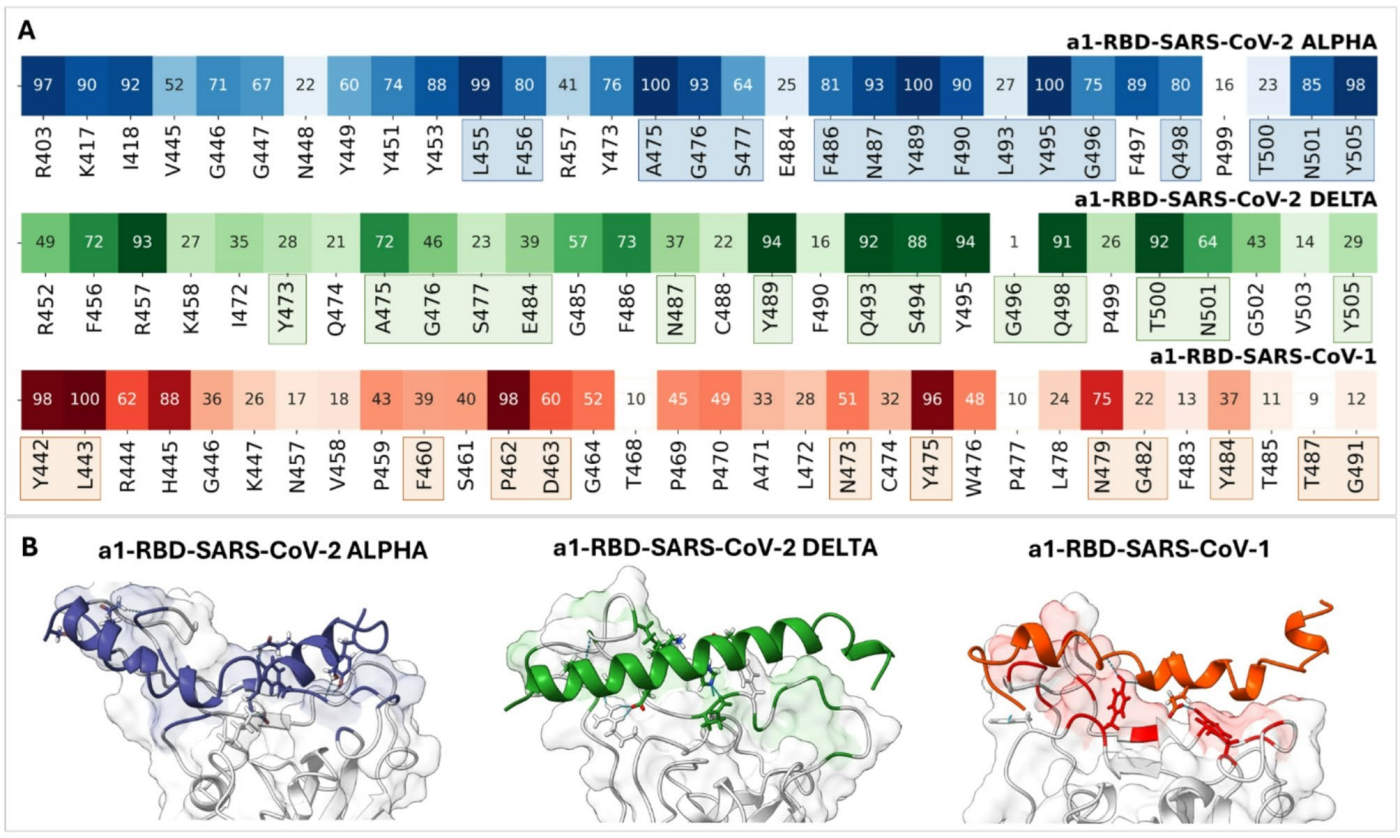
**A** Interaction heatmaps for complexes formed by **a1** and the RBD domains of SARS-CoV-2 Alpha, SARS-CoV-2 Delta, and SARS-CoV-1. Highlighted residues correspond to the recognition region for the natural receptor in the crystallographic structures 6M17, 2AJF and 7TEW, considering a distance cutoff of 5 Å. **B** Snapshots of the MD trajectories in which the recognition regions of the natural receptor are displayed as colored surfaces (blue: SARS-CoV-2 Alpha, green: SARS-CoV-2 Delta, red: SARS-CoV-1) and **a1** is represented as colored ribbons.

For **a1**, the only peptide that binds the RBDs in the region targeted by ACE2, we compared the intermolecular contact maps obtained from MD simulations with the structural models taken as reference in this study (6M17, 2AJF, and 7TEW). Heatmaps showing the frequency of **a1**-RBD contacts throughout the simulated MD trajectories are displayed in Fig. 6A. The residues that are part of the interaction site in the structural models (considering a distance cutoff of 5 Å) are highlighted under each heatmap and displayed in colored surfaces in Fig. 6B. The contact maps show that **a1** interacts with a conserved region of the RBD domains that is also involved in ACE2 recognition. For the Alpha variant, this region includes L455, A475, Y489, and Y495, which are relevant for the receptor’s binding to the virus. These findings highlight the potential of **a1** to mimic the intermolecular recognition properties of the natural receptor ACE2 toward b-type coronaviruses, which is a relevant outcome to support the design of biomimetic sensors based on this epitope.

**Fig. 7:**
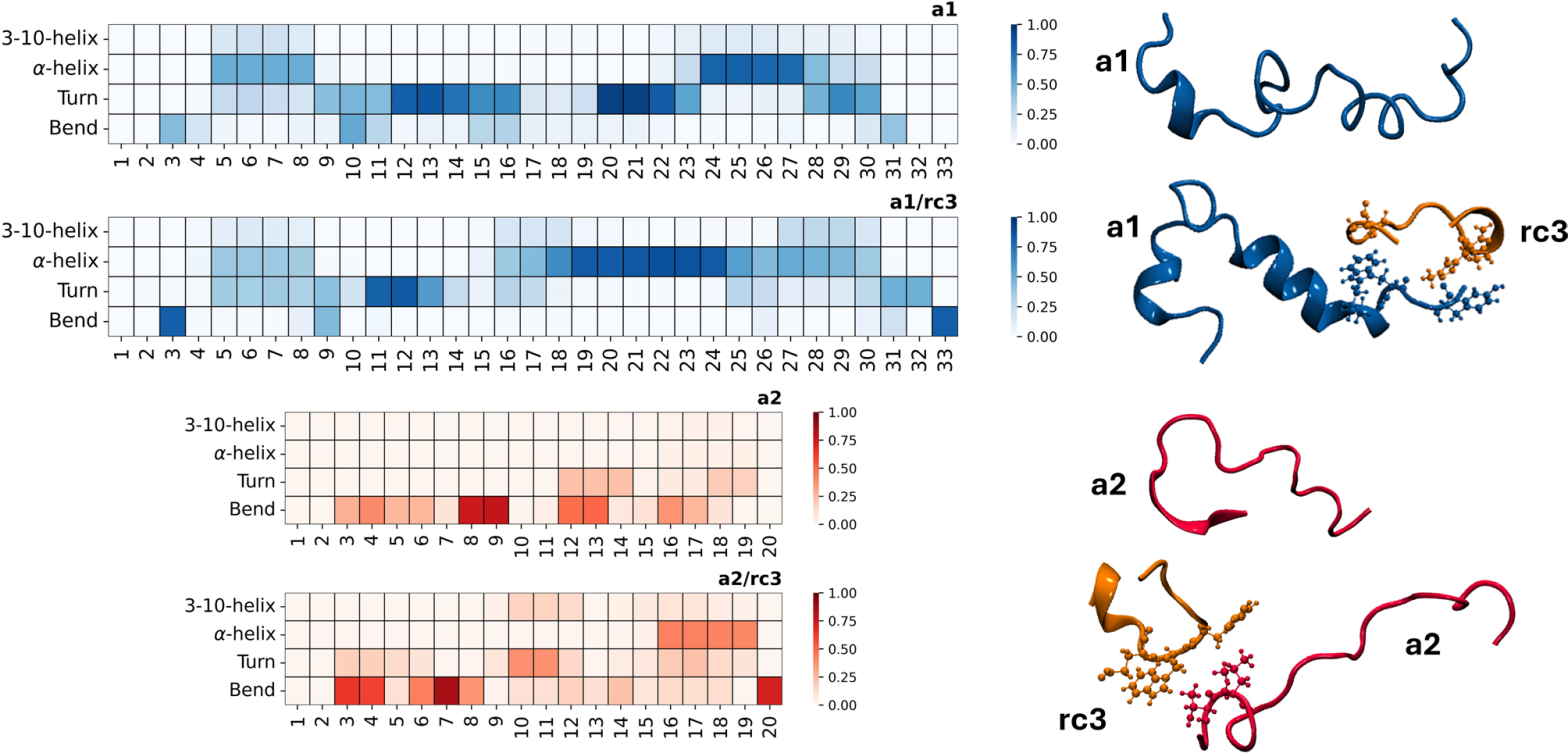
Analysis of secondary structures for **a1** and **a2** as individual peptides and in binary mixtures with **rc3**. Data was obtained from 500 ns MD simulations.

For our dual peptide mixtures, we first conducted MD simulations to examine potential peptide-peptide interactions that might occur on the surface of SPR receptors. We reasoned that **rc3** could not alter the recognition of the RBDs in dual mixtures due to its negligible individual binding capacity. Therefore, the observed variations in RBD affinity for the dual-peptide sensors might stem from a cooperative effect driven by the interaction between the peptides. While this is a tentative approach, our experimental setup allows us to assume that such interactions are indeed possible, given the length and flexibility of the polymer chains attached to the peptides and the high immobilization efficiency achieved for the dual-peptide sensors (Table 1). We investigated the potential interaction between peptides using MD simulations of binary **a1/rc3** and **a2/rc3** systems, comparing the behavior of the mixture to that of the individual peptides. Our results indicate that **rc3** interacts with both **a1** and **a2**, forming transient contacts that stabilize the α-helix secondary structures in the longer peptides (Fig. 7). This stabilizing effect is more pronounced in the **a1/rc3** system, particularly in the region spanning amino acids 16–30. This conformational restriction could affect the interaction with RBDs by either reducing the entropic cost of achieving the helical structure needed for an effective interaction with the recognition region or by favoring stabilizing/destabilizing contacts with the surface of the target protein.

**Fig. 8:**
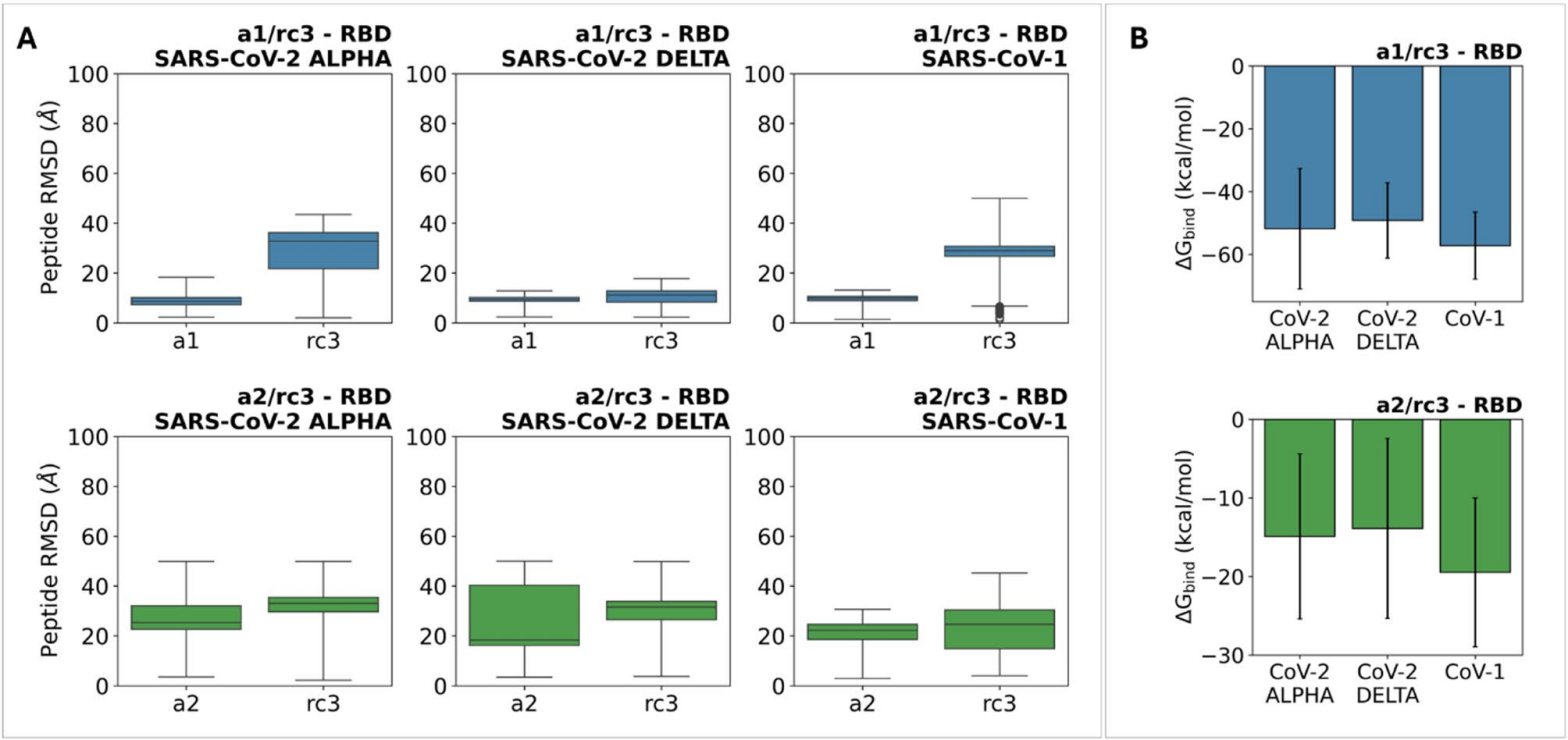
**A** RMSD distributions for **a1/rc3** and **a2/rc3** peptides throughout 500 ns MD trajectories in dual-peptide complexes with the RBD of SARS-CoV-2 Alpha, SARS-CoV-2 Delta, and SARS-CoV-1. **B** MM/GBSA binding free energy estimates (kcal/mol) for the interaction between the **a1/rc3** and **a2/rc3** mixtures with the RBD of SARS-CoV-2 Alpha, SARS-CoV-2 Delta, and SARS-CoV-1 calculated from 500 ns MD simulations.

To further attempt to understand the effect of the peptide mixture, we performed additional MD simulations on RBD complexes with the **a1/rc3** and **a2/rc3** peptide mixtures. The results show a marked reduction in peptide mobility compared to that observed in the corresponding single-peptide complexes, mediated by inter-peptide interactions on the RBD recognition region (Figure S5). The distribution of peptide RMSD in the dual complexes confirms this observation, suggesting a favorable effect on the stabilization of peptide-RBD contacts compared to complexes with isolated peptides (Fig. 8A). On the other hand, MM/GBSA binding free energies only suggest a moderate stabilization in the complex formed by the **a1/rc3** mixture and the SARS-CoV-1 RBD (Fig. 8B). For the other complexes, no significant variations in binding free energy were observed compared to single-peptide complexes. In this regard, it is worth noting that the MM/GBSA method only captures the enthalpic components of the peptide-RBD association. A more exhaustive approximation of the global recognition process would require addressing the entropic contribution. That said, the lower calculated interaction energy for the complex between **a1/rc3** and the SARS-CoV-1 RBD could account for a strengthening of the enthalpic terms responsible for RBD recognition. This could be further enhanced by a decrease in the entropic cost necessary for the peptides to adopt the required conformation and secondary structure for an effective interaction with the RBD’s recognition region.

## Conclusion

Frequently occurring mutations in virus surface proteins compromise first-generation antigen tests, leading to a loss or reduction of specificity and false-negative results. Moreover, infectivity relies not only on interactions with the main host cell receptor (ACE2) but also involves weakly binding co-receptors such as glycans. These weak co-receptors, insufficient alone to bind the virus with high affinity, manifest their role through multivalent interactions when acting in concert with a stronger receptor. An ideal host cell biomimetic should therefore provide a multivalent display of weak and strong receptors. This phenomenon aligns with the cooperative binding effects observed in this work, where the decapeptide **rc3**, which did not bind any of the variants as a single epitope, significantly contributed to both affinity and specificity when combined with epitopes of the main ACE2 contact region. MD simulations further supported this finding, showing that **rc3** promotes alpha-helix formation, leading to a more ACE2-like secondary structure.

Dual-epitope systems revealed remarkable enhancements in binding affinity and specificity across SARS-CoV-2 Alpha, and SARS-CoV-1 RBDs. For SARS-CoV-1, the dual sensors achieved a dramatic increase in binding affinity compared to single sensors, reversing the binding preference typically seen with isolated epitopes. This suggests that **rc3** may stabilize the structure of **a1**, promoting optimal interactions with the SARS-CoV-1 RBD. Similarly, the dual sensor containing **a2** showed substantial improvements in affinity and thermodynamic stability, narrowing the performance gap with **a1** and demonstrating the cooperative advantage of multivalent interactions. Additionally, both dual sensors exhibited superior selectivity for β-type coronaviruses, as evidenced by their rejection of the spike protein of HCoV-NL63, a mild α-coronavirus.

Interestingly, the full spike protein results revealed even stronger binding affinities compared to RBD-only systems, highlighting the additional binding opportunities provided by the multivalent and complex structure of the spike protein. This suggests that while dual sensors offer remarkable improvements in specificity and binding affinity, the full spike system further amplifies these effects, likely due to enhanced multivalency.

Taken together, these findings demonstrate that RBD affinity and variant specificity can be adjusted through a judicious choice of epitope combinations. By excluding sequences with disrupted contacts and including those promoting conformational stabilization or new contacts, tightly binding multivalent receptors can be rapidly engineered in response to emerging variants. This approach holds promise for developing robust diagnostic tools and therapeutics that adapt to evolving viral landscapes, ensuring both sensitivity and selectivity for β-type coronaviruses.

## Methods

### Reagents and equipment

Peptide epitopes containing a C-terminal propargylglycine (pra) were purchased from LifeTein. The FASTA sequences and the ACE2 residues corresponding to each epitope are: **a1** (STIEEQAKTFLDKFNHEAEDLFYQSSLASWNYN-pra-NH2), residues 19–52 (pl 4); **a2** (QSTLAQMYPLYEIYNLTVKL-pra-NH2), residues 76–95 (pI 6.5); and **rc3** (MTQGFWENSMLT-pra-NH2) residues 323–334 (pI 1–2). Hexapro spike protein with His-tag was kindly provided by SciLifeLab. SARS-CoV-2 RBD, SARS-CoV-1 RBD, and SARS-CoV-2 delta variant RBD, and the HCoV-NL63 spike protein were purchased from SinoBiological. All other reagents were purchased from Merck. Azide-modified sensor chips were purchased from Xantec and contained carboxylated dextran 3D matrix terminated by azide groups. Surface plasmon resonance (SPR) measurements were performed on a Biacore 3000 instrument, and the measurements were performed on the BIAevaluation program.

### Peptide immobilization on a gold sensor

An azide-modified SPR sensor chip (Xantec AZD 50 L) was inserted into the SPR instrument and equilibrated by running 5 µL/min HBS-T (10 mM HEPES, 150 mM NaCl, 0.005% Tween 20, pH 7.5) running buffer overnight. Click reaction mixtures of peptide (0.1 mM for **a1** and **rc3**, 0.05 mM for **a2**), sodium ascorbate (5.0 mM) and copper sulfate pentahydrate (1.0 mM) in either acetate buffer at pH 4 (**a1**), pH 6 (**a2**) or pH 2–3 (**rc3**) were prepared before the immobilization, the mixtures were filtered through 0.22 μm filters for sterilization and sonicated for 20 min to denature the peptides. Each reaction mixture was flowed over a separate channel on the surface while one channel was kept unmodified as a reference, at 5 µL/min using HBS-T as a running buffer while monitoring the change in response by SPR. The click reaction was followed by 2–3 pulses of 20 µL bicarbonate regeneration buffer (NaHCO_3_ and Na_2_CO_3_, 0.005% Tween 20, pH 9.5) followed by a 40 µL bicarbonate regeneration buffer (NaHCO_3_ and Na_2_CO_3_, 1 M NaCl, pH 9.5) to remove excess peptide from the surface. The process was repeated until a satisfactory immobilization level (more than 100 RU change) was reached.

### Surface plasmon resonance (SPR)

The peptide-functionalized SPR sensor chip was equilibrated by running 30 µL/min HBS-T (10 mM HEPES, 150 mM NaCl, 0.005% Tween 20, pH 7.5) running buffer for a minimum of 30 min followed by 20 µL pulses of the running buffer until a stable baseline was obtained (< 0.5 RU/min). The SARS-CoV-2 RBD or spike protein was serially diluted 2-fold from 100 nM to 1.5625 nM (RBD) or 25 nM to 3.125 nM (spike protein) in the HBS-T running buffer. The kinetic interaction analysis was performed in the multicycle mode by injecting 150 µL of the six dilution samples into all surface channels at 30 µL/min in the order from lowest to highest concentration, followed by 300 s dissociation time after each injection. The kinetic data was fitted globally using the BIAevaluation software (v.4.1) and a kinetic titration model provided by Karlsson et al.^12^ Each protein analysis was followed by 2–3 pulses of 90 µL regeneration bicarbonate buffers to remove protein from the surface, followed by 90 µL pulses of HBS-T running buffer until a stable baseline was obtained. The surface chip was unmounted from the SPR instrument only after all analyses had been performed.

### Molecular dynamics simulations

The initial coordinates of the SARS-CoV-2 RBD domain and peptides **a1**, **a2**, and **rc3** were obtained from the cryo-electron microscopy structure of the complex between the receptor binding domain of the SARS-CoV-2 Spike protein and the angiotensin-converting enzyme 2 (ACE2) released under the PDB code 6M17 (2.90 Å resolution)^9^. Peptides **a1**, **a2**, and **rc3** correspond to fragments 19–52, 76–95, and 323–334 of the ACE2 protein, respectively. The peptides were modified at the C-terminus with a heterocyclic linker moiety that mimics the connectivity of the peptide to the SPR sensor (Figure S3). AMBER-like force field parameters for this linker group were obtained using the ANTECHAMBER software with charges calculated at the AM1-BCC level, which is a fast, accurate, and robust alternative to HF/6-31G ESP-fit charges for general use with the AMBER force field in computer simulations involving small organic molecules^23^. The initial coordinates of the RBD domains of the β-type variants SARS-CoV-1 and SARS-CoV-2 Delta were obtained from the structural models of the corresponding complexes with their receptors released under PDB codes 2AJF and 7TEW^24,25^. The missing segments of the RBD domains were built by homology modeling using the PDB models 7WR9 and 7V8B as reference, respectively. The RBD variant domains were aligned with the SARS-CoV-2 RBD structure using the pairwise structure alignment tool available on the Protein Data Bank platform. The initial coordinates of the RBD of the α-corona virus HCoV-NL63 were obtained from the PDB model 3KBH, completed by homology modeling using the structure released under PDB code 7FC3, and aligned with the SARS-CoV-2 Alpha RBD^14^. The protonation states of the titratable residues of the protein and peptides under study were set to pH 7.4 using the ProteinPrepare web tool^26^. The N- and C-termini of the RBD domains were capped with acetyl and N-methyl amido groups. The peptide-RBD complexes were constructed using the LEAP software available in the AMBER21 program^27^, using the ff19SB force field parameters for protein and peptide moieties^28^. The complexes were neutralized with Na + and Cl- ions and solvated with explicit OPC water molecules in a cubic box that extends 15 Å from the outermost atoms of each system in all three dimensions. Molecular dynamics simulations were performed following an experimental protocol that includes the following steps: (a) 5000 steps of steepest descent minimization plus 3000 steps of conjugate gradient minimization for water relaxation, (b) 5000 steps of steepest descent minimization followed by 3000 steps of conjugate gradient minimization for the entire system, (c) 1 ns of progressive NVT heating from 0 to 300 K, (d) 500 ps of NPT density equilibration, (e) 20 ns of NPT equilibration at 300 K, and (f) 500 ns of NPT production dynamics at 300 K and 1 bar from which production data were collected. MD simulations used a 10 Å cutoff for non-bonded energy terms, bonds involving hydrogen atoms were constrained using the SHAKE algorithm, and long-range electrostatics were treated using the Particle-Mesh Ewald approach. Throughout the simulation, the acetyl and N-methylamide groups of the N- and C-terminal segments of the RBD domains were kept restrained with a force constant of 10 kcal mol^−1^ Å^−2^. The molecular dynamics trajectories were analyzed using the CPPTRAJ^29^ and VMD^30^ software. MM/GBSA simulations were performed to estimate the binding free energy for the interaction between the peptides and each RBD, using a single trajectory approach and a salt concentration of 0.15 M.^31^

## Supplementary Information

Below is the link to the electronic supplementary material.

Supplementary Material 1

## Data Availability

The datasets generated and/or analysed during the current study are available in the github repository, https://github.com/verjimenezc/Dujaili\_2025.

## Abbreviations

ACE2: Angiotensin-converting enzyme 2
K_D_: Dissociation constant
pI: Isoelectric point
RBD: Receptor binding domain
RU: Response units
RU_max_: Maximum response units
SPR: Surface plasmon resonance
