# Supplementary material for: Dual ACE2 epitope-based biomimetic receptors for selective sensing of SARS-CoV variants
Source: Sci Rep. 2025 Sep 23;15:32687. doi: 10.1038/s41598-025-20837-6 (PMC12457685; doi:10.1038/s41598-025-20837-6)
Supplement: Supplementary file 1 — Supplementary Material 1 [file 41598_2025_20837_MOESM1_ESM.pdf]

Supplementary Information

Dual ACE2 Epitope-Based Biomimetic Receptors for Selective Sensing of SARS-CoV Variants

Tiba Al-Dujaili[a], Sara Björk Sigurdardóttir[a], Verónica A. Jiménez[b], Michele Larocca[a], Börje Sellergren[a]

[a] Department of Biomedical Sciences and Biofilms-Research Center for Biointerfaces (BRCB), Faculty of Health and Society, Malmö University, 20506 Malmö, Sweden.

[b] Departamento de Ciencias Químicas, Facultad de Ciencias Exactas, Universidad Andres Bello, Autopista Concepción-Talcahuano 7100, Talcahuano, Chile.

Table of Contents

1. **ACE2 Epitopes and Their Interactions with Covid Variants** 2
2. **SPR-Based Kinetic Interaction Analysis** 4
3. **Molecular Dynamics Simulations** 8
4. **References** 10

1. ACE2 Epitopes and Their Interactions with Covid Variants

Table S1. ACE2 epitope proper3es and pep3de sequence informa3on

Epitope	PI	Mw (g/mol)	Sequence
**a1**	4	4021	19-STIEEQAKTFLDKFNHEAEDLFYQSSLASWNYN-52
**a2**	6.5	2483	76-QSTLAQMYPLYEIYNLTVKL-95
**rc3**	1	1538	323-MTQGFWENSMLT-334

Table S2. Cumulative number of Van der Waals and hydrogen bond contacts (underlined) between the ACE2 a1 epitope and Covid variants based on published Cryo-EM data.[1-3]

ACE2 amino acid residue	SARS-CoV-1	SARS-CoV-2-prototype	SARS-CoV-2 Delta
S19	1	7,1	3
Q24	6,**1**	24,**1**	13,**1**
T27	8	15	13
F28	7	7	8
D30	2	10	10
K31	12	19	14,**1**
H34	10,**1**	20,**1**	13,**2**
E35	-	8	-
E37	4	7	7,**1**
D38	11,**2**	15,**1**	11,**1**
Y41	25,**1**	23,**2**	28,**1**
Q42	9,**1**	16,**5**	15,**3**
L45	3	4	2

Table S3. Cumula3ve number of Van der Waals and hydrogen bond contacts (underligned) between the ACE2 a2 epitope and Covid variants based on published Cryo-EM data.[1-3]

ACE2 amino acid residue	SARS-CoV-1	SARS-CoV-2-prototype	SARS-CoV-2 Delta
L79	2	2	-
M82	4	9	7
Y83	10,**2**	20,**1**	18,**2**

Table S4. Cumulative number of Van der Waals and hydrogen bond contacts (underlined) between the ACE2 rc3 epitope and Covid variants based on published Cryo-EM data.[1-3]

ACE2 amino acid residue	SARS-CoV-1	SARS-CoV-2-prototype	SARS-CoV-2 Delta
Q325	4	-	-
E329	6,**1**	-	-
N330	11,**1**	8	4

2. SPR-Based Kinetic Interaction Analysis

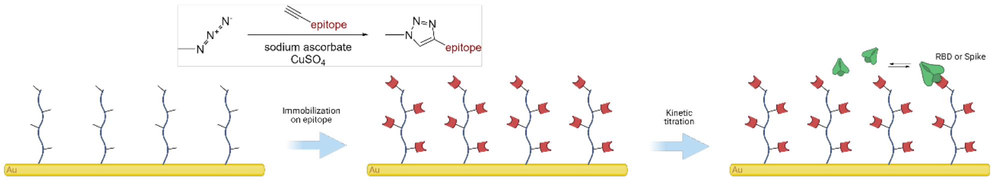

Figure S1. Schematic representation of the epitope immobilization to a carboxymethylated dextran SPR chip via a Huisgen type cycloaddition reaction.

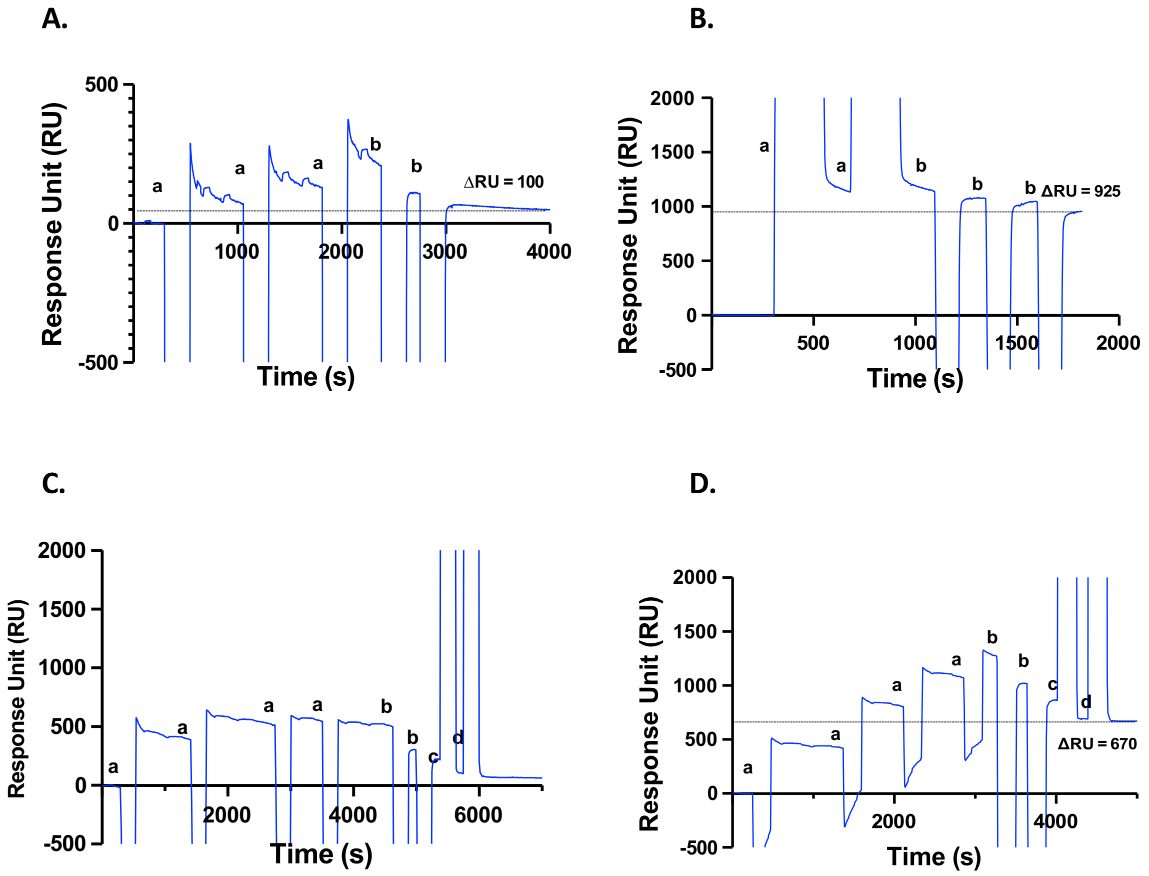

Figure S2. SPR sensograms monitoring the immobilization of A) a1, B) a2, C) a1/rc3 and D) a2/rc3 by click coupling. a) Addition of the epitope click reaction mixture; b) bicarbonate regeneration buffer; c and d) bicarbonate regeneration buffer including 1M NaCl.

Table S5. Single and dual epitope immobilization results are expressed in two ways: change in response unit (ΔRU) and number of immobilized molecules per mm² (N) of the epitopes, as determined from two different sets of experiments on two different SPR chips.

Epitope	Chip #	M_w (g/mol)	m_{mol} x 10^{21} (g) [a]	ΔRU	m_{immob} x 10^{10} (g/mm²) [b]	N x 10^{-10} [c]
**a1**	1	4021	6.68	175	1.75	2.61
**a1**	2	4021	6.68	100	1.00	1.49
**a2**	1	2483	4.12	925	9.25	22.5
**a2**	2	2483	4.12	834	8.34	20.2
**rc3**	1	1538	2.55	397	3.97	15.6
**rc3**	2	1538	2.55	217	2.17	8.51
**a1/rc3**	1	2779 [d]	4.61	1170	11.70	25.4
**a1/rc3**	2	2779 [d]	4.61	433	4.33	9.39
**a2/rc3**	1	2010 [d]	3.34	430	4.30	12.9
**a2/rc3**	2	2010 [d]	3.34	669	6.69	20.0

a) m_{mol} = mass per molecule = M_w/N_A (N_A=Avogadro number)
b) m_{immob} = mass immobilized = ΔRU x 10^{-12} g/mm²
c) N = number of immobilized molecules per mm² = m_{immob} / m_{mol}
d) Weighted average mass of the two epitopes.

Table S6. Evaluation of fit metrics (χ^2, RMSE, R^2) for sensogram fitting of Alpha, Delta, and SARS-CoV-1 for all sensors.

RBD variant	Epitope	Chi²	RMSE (RU)	R²
SARS-CoV-1	**a1**	0,94	2,4	0,94
	**a2**	1,8	1,8	0,86
	**a1+rc3**	1,48	2,1	0,89
	**a2+rc3**	1,75	1,7	0,97
SARS-CoV-2 Alpha	**a1**	1,1	1,3	0,99
	**a2**	1	1,6	0,94
	**a1-rc3**	0,65	1,8	0,90
	**a2-rc3**	9	6,2	0,64
SARS-CoV-2 Delta	**a1**	1,68	2	0,91
	**a2**	1,35	2,6	0,89
	**a1+rc3**	0,47	1	0,99
	**a2+rc3**	2,53	2,4	0,70

Table S7. Results from individual measurements using SPR-based kinetic interaction analysis of the interaction between β-type corona viral variants (SARS-CoV-2 Alpha, SARS-CoV-2 Delta, and SARS-CoV-1) RBDs and the single epitope sensors based on a1 and a2.

RBD variant	Epitope	k_a ($M^{-1}s^{-1}$)	k_d (s^{-1})	K_D (M)
SARS-CoV-1	**a1**	7,92e4	4,49e-3	5,67e-8
	**a1**	8,85e4	4,5e-3	5,09e-8
	**a1**	8,35e4	4,84e-3	5,8e-8
	**a2**	8,59e4	2,18e-3	2,54e-8
	**a2**	1,04e5	2,33e-3	2,23e-8
	**a2**	1,43e5	2,08e-3	1,46e-8
	**rc3**	5,32e3	9,97e-3	1,87e-6
	**rc3**	2,50e3	8,20e-3	3,28e-6
SARS-CoV-2 Alpha	**a1**	1,62e5	4,11e-3	2,54e-8
	**a1**	1,45e5	5,24e-3	3,62e-8
	**a1**	1,57e5	3,98e-3	2,53e-8
	**a2**	5,93e4	5,06e-3	8,53e-8
	**a2**	5,66e4	5,28e-3	9,32e-8
	**a2**	6,08e4	8,05e-4	1,32E-8
	**rc3**	8,39e3	2,68e-3	3,20e-6
	**rc3**	7,83e3	5,11e-3	6,53e-6
SARS-CoV-2 Delta	**a1**	1,96e5	3,28e-3	1,67e-8
	**a1**	1,87e5	3,07e-3	1,65e-8
	**a1**	1,69e5	2,6e-3	1,54e-8
	**a2**	6,04e4	3,15e-3	5,21e-8
	**a2**	5,98e4	3,79e-3	6,34e-8
	**a2**	6,12e4	3,27e-3	5,34e-8
	**rc3**	1,83e3	9,97e-3	5,44e-6
	**rc3**	4,43e3	2,64e-3	5,97e-6

Table S8. Results from individual measurements using SPR-based kinetic interaction analysis of the interaction between SARS-CoV-2 Spike protein and the single epitope sensors based on a1 and a2.

Epitope	k_a ($M^{-1}s^{-1}$)	k_d (s^{-1})	K_D (M)
**a1**	1,25e5	2,02e-4	1,62e-9
**a1**	1,34e5	1,47e-4	1,1e-9
**a1**	5,94e4	5,02e-5	8,46e-10
**a2**	7,39e4	2,42e-3	3,28e-8
**a2**	9,98e4	1,4e-3	1,4e-8
**a2**	1e5	1,19e-3	1,18e-8
**rc3**	7,5e3	1,53e-3	2,04e-6
**rc3**	6,2e3	6,386e-3	1,03e-5

Table S9. Results from individual measurements using SPR-based kinetic interaction analysis of the interaction between β-type corona viral variants (SARS-CoV-2 Alpha, SARS-CoV-2 Delta, and SARS-CoV-1) RBDs and the dual epitope sensors based on a1, a2 and rc3.

RBD variant	Epitope	k_a ($M^{-1}s^{-1}$)	k_d (s^{-1})	K_D (M)
SARS-CoV-1	**a1+rc3**	1,06e5	7,17e-4	6,75e-9
	**a1+rc3**	1,37e5	5,74e-4	4,2e-9
	**a1+rc3**	1,01e5	7,78e-4	7,67e-9
	**a2+rc3**	1,69e5	3,17e-3	1,87e-8
	**a2+rc3**	1,27e5	3,06e-3	2,4e-8
	**a2+rc3**	1,8e5	3,55e-3	1,97e-8
SARS-CoV-2 Alpha	**a1+rc3**	1,61e5	2,45e-3	1,53e-8
	**a1+rc3**	1,55e5	2,77e-3	1,79e-8
	**a1+rc3**	1,54e5	2,62e-3	1,7e-8
	**a2+rc3**	8,84e4	1,76e-3	1,99E-8
	**a2+rc3**	8e4	1,82e-3	2,27E-8
	**a2+rc3**	8,04e4	1,89e-3	2,35e-8
SARS-CoV-2 Delta	**a1+rc3**	9,05e4	3,37e-3	3,73e-8
	**a1+rc3**	9,23e4	3,81e-3	4,13e-8
	**a1+rc3**	9,08e4	3,58e-3	3,94e-8
	**a2+rc3**	1,01e5	1,92e-3	1,9e-8
	**a2+rc3**	9,13e4	2e-3	2,19e-8
	**a2+rc3**	9,11e4	1,86e-3	2,04e-8

3. Molecular Dynamics Simulations

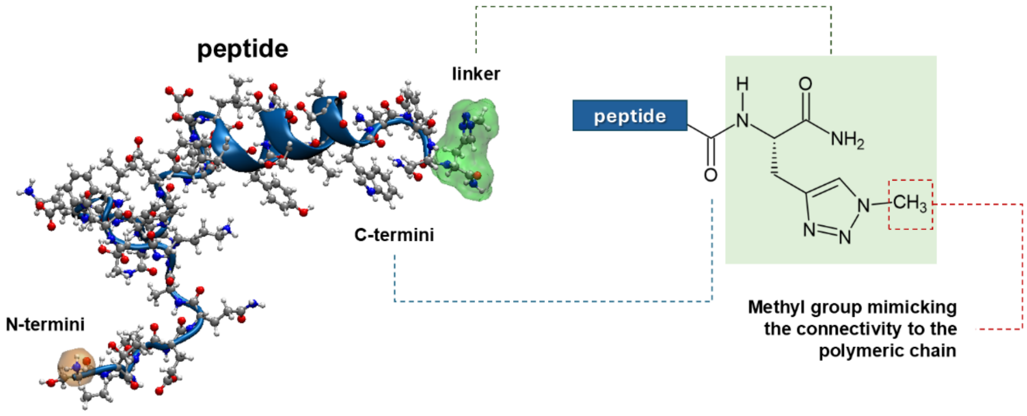

Figure S3. Representative structure of a peptide modified at the C-termini with a linker moiety that mimics the connectivity to the polymeric chain in the SPR sensor.

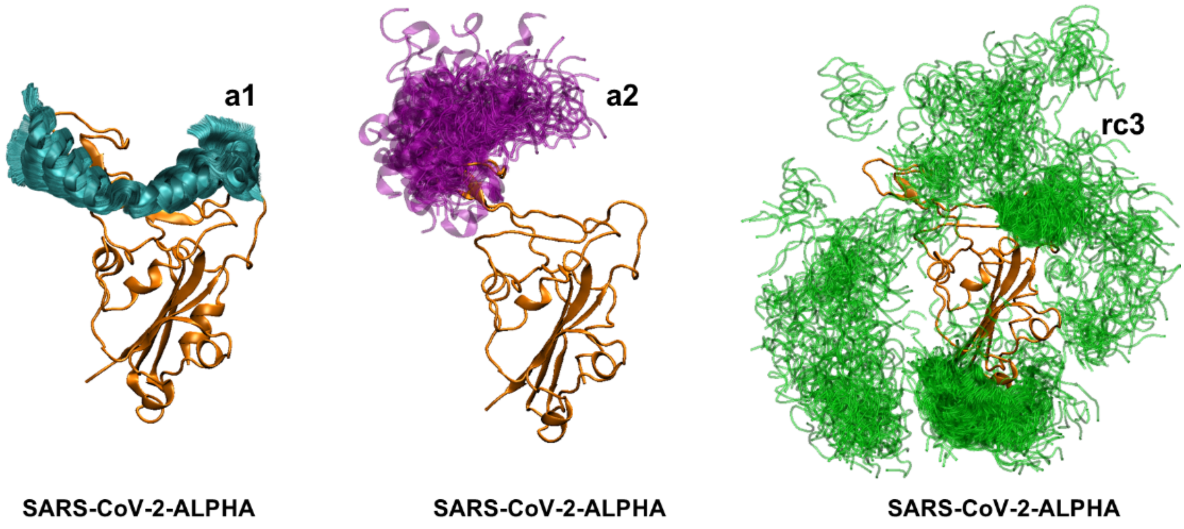

Figure S4. Representation of the time evolution of SARS-CoV-2-Alpha RBD complexes with peptides a1, a2 and rc3 taken from 500 ns MD simulations.

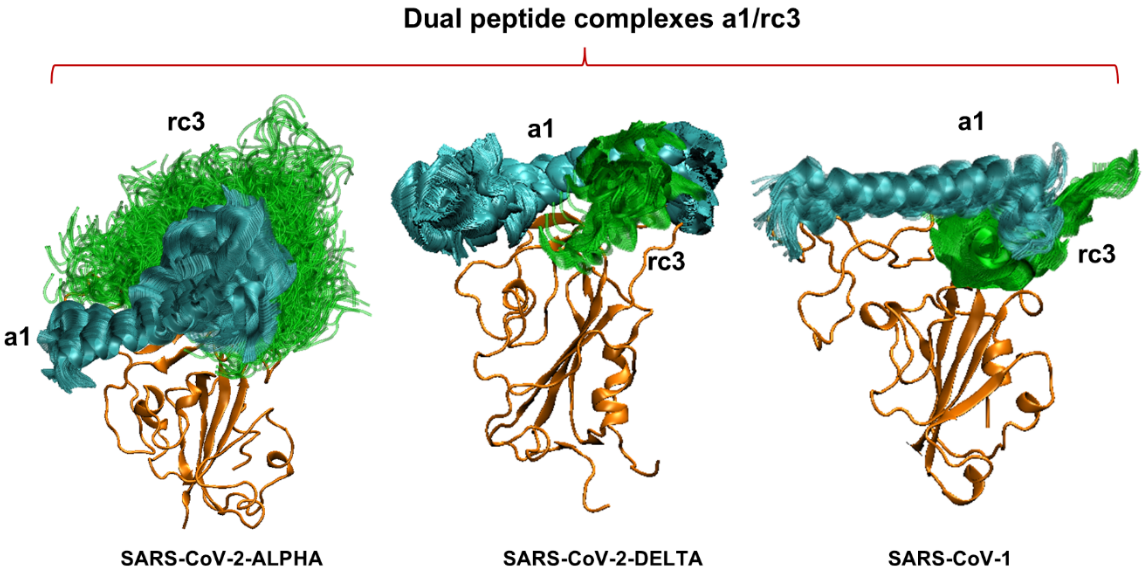

Figure S5. Representation of the time evolution of dual peptide complexes between **a1/rc3** and the RBDs of SARS-CoV-2 Alpha, SARS-CoV-2 Delta and SARS-CoV-1, taken from 500 ns MD simulations. The results show that the dual complexes have a significant reduction in peptide mobility compared to complexes with individual peptides. We also observed that the **a1** peptide preserves its alpha-helical secondary structures due to a cooperative interaction with **rc3**.

Table S10. MM/GBSA binding free energy estimates (kcal/mol) obtained for single- and dual-peptide complexes with RBD variants.

RBD	Peptide(s)	Binding energy (kcal/mol)	Standard Deviation (kcal/mol)	Standard Error (kcal/mol)
**SARS-CoV-2 Alpha**	a1	-59.3	10.9	0.5
**SARS-CoV-2 Delta**	a1	-58.5	11.4	0.7
**SARS-CoV-1**	a1	-43.3	14.2	0.6
**SARS-CoV-2 Alpha**	a1/rc3	-51.8	19.2	0.4
**SARS-CoV-2 Delta**	a1/rc3	-49.2	11.9	0.3
**SARS-CoV-1**	a1/rc3	-57.1	10.7	0.4
**SARS-CoV-2 Alpha**	a2	-19.1	4.0	0.2
**SARS-CoV-2 Delta**	a2	-24.0	9.2	0.6
**SARS-CoV-1**	a2	-27.9	10.9	0.5
**SARS-CoV-2 Alpha**	a2/rc3	-14.9	10.5	0.2
**SARS-CoV-2 Delta**	a2/rc3	-13.9	11.5	0.3
**SARS-CoV-1**	a2/rc3	-19.5	9.5	0.2

4. References

(1) Wang, Q.; Zhang, Y.; Wu, L.; Niu, S.; Song, C.; Zhang, Z.; Lu, G.; Qiao, C.; Hu, Y.; Yuen, K.-Y. Structural and functional basis of SARS-CoV-2 entry by using human ACE2. Cell 2020, 181 (4), 894-904. e899.

(2) Rossi, G. A.; Sacco, O.; Mancino, E.; Criseani, L.; Midulla, F. Differences and similarities between SARS-CoV and SARS-CoV-2: spike receptor-binding domain recognieon and host cell infection with support of cellular serine proteases. Infection 2020, 48 (5), 665-669.

(3) Dejniralsai, W.; Huo, J.; Zhou, D.; Zahradník, J.; Supasa, P.; Liu, C.; Duyvesteyn, H. M.; Ginn, H. M.; Mentzer, A. J.; Tuekprakhon, A. SARS-CoV-2 Omicron-B. 1.1. 529 leads to widespread escape from neutralizing antibody responses. Cell 2022, 185 (3), 467-484. e415.

(4) Karlsson, R.; Katsamba, P. S.; Nordin, H.; Pol, E.; Myszka, D. G. Analyzing a kinetic titration series using affinity biosensors. Analytical Biochemistry 2006, 349 (1), 136-147.
